# Alerta Cannabis: A Tailored-Computer Web-Based Program for the Prevention of Cannabis Use in Adolescents: A Cluster-Randomized Controlled Trial Protocol

**DOI:** 10.1186/s12912-024-01889-x

**Published:** 2024-04-10

**Authors:** Marta Lima-Serrano, Carmen Barrera-Villalba, Isotta Mac-Fadden, Ilse Mesters, Hein de Vries

**Affiliations:** 1 Instituto de Biomedicina de Sevilla, IBiS. Department of Nursing, School of Nursing, Physiotherapy and Podiatry, University of Seville, Seville, Spain; 2 Department of Health Promotion, Care and Public Health Research Institute CAPHRI, Maastricht University, Maastricht, The Netherlands; 3 Department of Sociology, University of Salamanca, Salamanca, Spain; 4 Department of Epidemiology, Care and Public Health Research Institute CAPHRI, Maastricht University, Maastricht, The Netherlands; 5 Department of Health Promotion, Care and Public Health Research Institute CAPHRI, Maastricht University, Maastricht, The Netherlands

**Keywords:** Cannabis use, Cluster randomized controlled trial, Adolescents, Web-based interventions, Computer-tailoring

## Abstract

**Background:**

The growing use of cannabis in adolescence is a public health problem that must be addressed through prevention. In Spain, the average age of initiation of cannabis use in the adolescent population is 14.8 years. At 14 years, the lifetime prevalence of cannabis use is 11.7%, which increases to 51.,5% at the age of 18; the prevalence of cannabis use in the population aged 14 to 18 years is 28.6%, a figure that must be tried to reduce, that is why this school prevention program is proposed: Alerta Cannabis.

**Methods:**

The Alerta Cannabis research project consists of design, implementation, and evaluation. In the first phase, a computer-tailored eHealth program (Alerta Cannabis) is developed based on the I-Change Model, an integrated model based on three main behavioral change processes: awareness, motivation, and action. This program consists of four 30-minute sessions that will provide culturally adapted and personalized advice to motivate students not to use cannabis through text feedback, animations, and gamification techniques. This phase will also include usability testing. In the implementation phase, secondary school students from Western Andalusia, Spain (Seville, Cádiz, Huelva, and Córdoba) and Eastern Andalusia (Jaén, Málaga, and Granada) will be randomized to an experimental condition (EC) or a control condition (CC) for a cluster randomized clinical trial (CRCT). Each condition will have 35 classes within 8 schools. GI will receive the online intervention Alerta Cannabis. EC and CC will have to fill out a questionnaire at baseline, six months, and twelve months of follow-up. In the last phase, the effect of Alerta Cannabis is evaluated. The primary outcomes are the lifetime prevalence of cannabis use and its use in the last 30 days and at 6 months. At 12 months of follow-up, the prevalence in the last 12 months will also be assessed. The secondary outcome is the intention to use cannabis.

**Discussion:**

The study tests the effect of the innovative program specifically aimed to reduce the use of cannabis in the adolescent population through eHealth in Spain. The findings aim to develop and implement evidence-based cannabis prevention interventions, which could support school prevention, for instance, the assistance of school nurses. If the program proves to be effective, it could be useful to prevent cannabis use on a national and international scale.

**Trial registration:**

NCT05849636. Date of registration: March 16, 2023.

## Introduction

Cannabis is the most widely consumed illegal drug in the world and with the general populations and adolescent populations in Europe, Spain, and Andalusia. According to the World Drug Report of the United Nations Office on Drugs and Crime [1], 209 million people used cannabis in the previous year. Furthermore, cannabis consumption is increasing; globally, the number of cannabis users has increased by 23% in the last decade. Cannabis use is more common among 15 to 16-year-olds (5.8%) than in the general population (4.1%). In Spain [2–4], the average age of initiation of cannabis use in the adolescent population is 14.8 years. At 14 years of age, the lifetime prevalence of cannabis use is 11.7%, which quintuples to 51.5% at 18 years old; the prevalence of cannabis use in the population aged 14 to 18 years is 28.6%. Among those under 18 years of age, cannabis is consolidated as the substance that generates the highest treatment admissions (95.1%).In Andalusia [3, 5], the lifetime prevalence of cannabis use in the population aged 14 to 18 is 21.4%, and cannabis use is the leading cause of treatment admissions for substance use in the adolescent population (86%). Recent scientific literature concludes that there is insufficient evidence regarding the association between cannabis use and all-cause mortality [6, 7]. However, some adverse health outcomes may be elevated among heavy cannabis users, such us fatal motor vehicle accidents, and possibly respiratory and brain cancers [6]. Furthermore, cannabis use disorder is a common comorbidity and risk marker for self-harm, mortality, and death by unintentional overdose and homicide among youth with mood disorders [8]. In fact, cannabis use is the drug associated with the highest number of disorders related to mental health [9].

A meta-analytic review [10] by Porath-Waller and Cols (2010) concluded that school-based programs have a positive impact on reducing adolescents‘ (aged 12–19) cannabis use compared to control conditions. Their results also suggested that targeting high school students is more effective than targeting middle school students. In Spain, the Ministry of Health has a system of health promotion and prevention that includes school-based health education in schools [11]. This is linked to the Strategy for Health Promotion and Prevention of the National System of Health. This includes a website for healthy lifestyles, a map of resources for health and the Information System of Health Promotion and Education (SIPES), where it is possible to publish actions that are carried out by different organizations at regional and national levels. Furthermore, a portal to include Good Practices for the reduction of demands for substance use and other addictions [12], with the objective of promoting the quality of interventions in Spain, that is to identify, select, transfer, and disseminate good practices in addictions. However, a review of school programs for substance abuse prevention in Spain indicated promising results although a lack of more rigorous evaluation is detected [13]. Moreover, a recent review concludes that most of the programs include in Spanish best practices portals did not evaluate their efficacy, while there are programs that having had their results evaluated are not indexed in said portals. Although Spain goes in the right direction, an evidence-based prevention model coexists with a pseudo-preventive model. Therefore, it is necessary to continue to promote a culture of evidence-based prevention and having efficacy evaluation protocols in prevention programs [14]. This review recommends the programs due to their strong evidence the Project EX and the Unplugged program. The last is a comprehensive program for substance use prevention based on the social influence approach and showed a reduction in the prevalence of cannabis. However, the data were not disaggregated by countries (the study was carried out in Seven European countries: Austria, Belgium, Germany, Greece, Italy, Spain, and Sweden) [15]. Moreover, in northern Spain, a universal program on drug use in general [16], ‘Be yourself’, showed a positive impact on the reduction of cannabis use in middle schools (aged 12–14) exposed to the intervention. The latter is based on models of social influence and social competence.

Research indicates that cannabis prevention programs can be effective if they use social cognitive models to convince adolescents of their disadvantages, how to deal with social influences that promote their use, and to increase self-efficacy and refusal skill [10, 17–19]. They also concluded that programs that incorporated elements of several prevention models were significantly more effective than those based solely on a social influence model [18, 19]. In addition, eHealth methods are also effective for cannabis treatment and have even better results for prevention [17]. In this line and view of the results of other investigations, the cultural compatibility of the prevention interventions must also be considered [20–23].

Based on this scientific evidence the school-based program Alerta Cannabis will be developed. It will use as theoretical model the Integrated Change Model [24], which has also turned out to be an explanatory model for the factors associated with the use of cannabis among Andalusian adolescents [25]. This model integrates existing social cognitive models and can be used as a basis in computer-tailored information and communication technology.

The starting hypothesis is that the application of this program, Alerta Cannabis, to minors between the ages of 14 and 18 in the school context, will be effective in reducing the prevalence of cannabis use. The objective of this study is to evaluate the effect of the Alerta Cannabis program, for which we will consider, as primary outcomes, different patterns of cannabis use such as cannabis use sometimes in life, in the last 30 days and the last 12 months, and, as a secondary outcome, the intention to use cannabis.

### The integrated change model

**Fig. 1:**
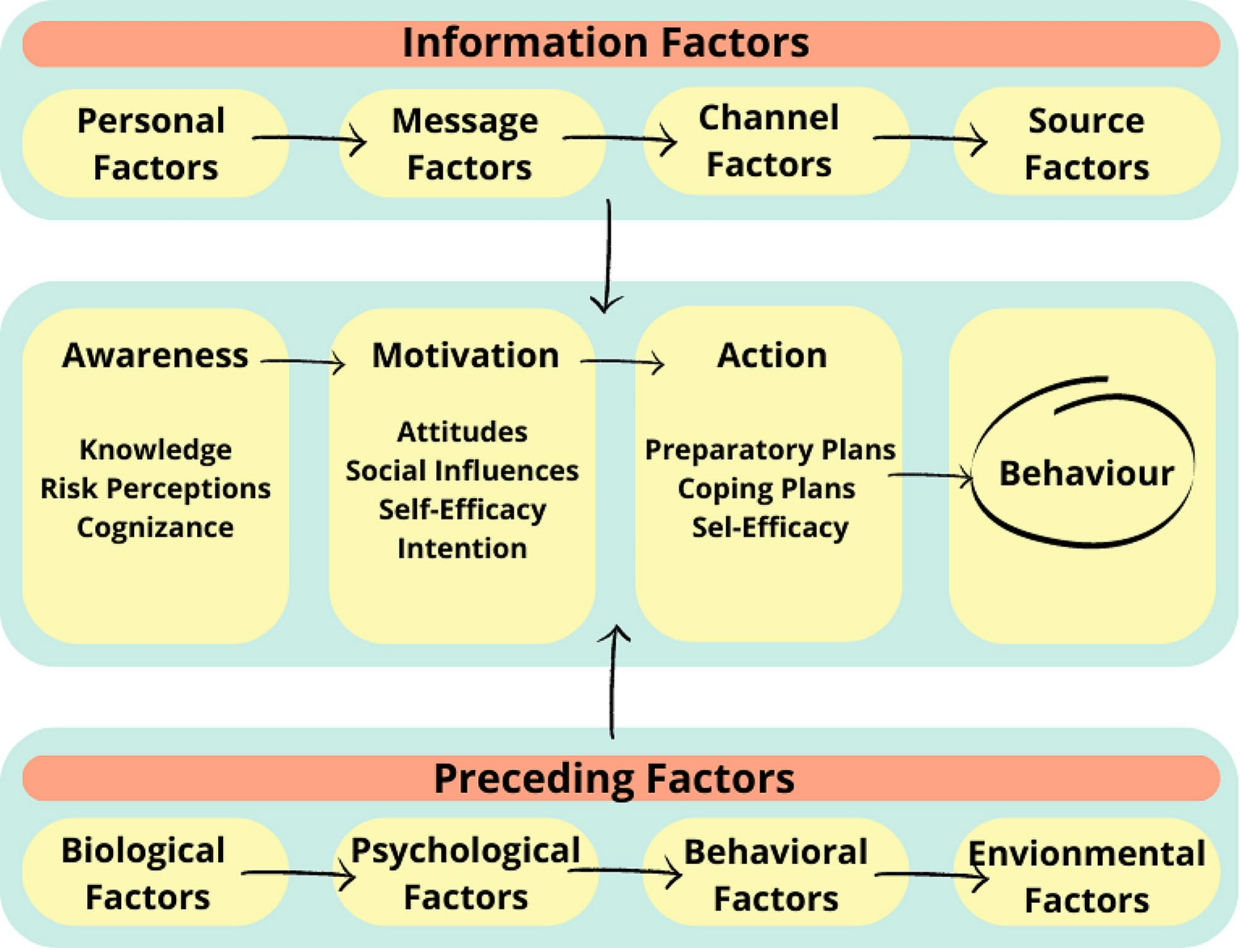
I-change model (De Vries, 2017)

The I-Change model is a behavior change model that tries to generate motivational and behavioral change in individuals based on their intentions and abilities [24]. The I-Change model integrates various theoretical models such as the Attitude Model- Social Influence-Self efficacy (or ASE-model) [26], the Theory of Planned Behavior by Ajzen [27, 28], Bandura’s Social Cognitive Theory [29], Transtheoretical Model of Change [30], Belief Model on Health and Goal Setting [31]. Behavior change is generated through three stages: pre-motivational, motivational, and post-motivational, with the motivational stage serving as the foundation [25]. Pre-motivational factors influence behaviors and consist of predisposing factors (behavioral, psychological, biological, social, and cultural), awareness factors (knowledge, cues to action, and risk perception), and information factors (message, channels, and source), which. Motivational factors, facilitate or condition action. They include attitude (advantages and disadvantages), social influence (social norm, social pressure, social model, support), and self-efficacy (barriers, emotions, and abilities), while post-motivational factors are related to skills (implementation plans, development skills, and actions objectives) (Fig. 1), which are key to convert intentions into actions [32, 33].

### Computer tailoring technology

Computer-tailoring technology can be defined as the process of adjusting intervention materials to the specific characteristics of an individual through a digitized process [34]. Unlike more static online communication, these interventions provide people with only the information that is relevant to them, as it is often personalized and tailored to the demographic characteristics and specific situations of the participants. As a result, this information is more likely to be considered relevant by the individual and, consequently, to be read [35]. This methodology provides individuals with personalized feedback on risk behaviors through messages adapted to their specific needs of the user while protecting their anonymity [34].

Web-based computer-tailored interventions (WBCT) are cost-effective for a variety of health-related behaviors in the adult and adolescent populations [36–38]. These interventions also have the potential to reach a large proportion of the adolescent population, since in Spain 99,7% of young people are Internet users, with hardly any differences by gender and/or social status [39]. WBTCT interventions improve young people’s accessibility, as they do not have space-time constraints, they generate personalized messages based on the motivational characteristics of participants; can attract the attention of individuals, and improve the processing of transmitted information through multiple senses [40, 41].

As a theoretical and methodological framework, the I-Change model has been applied in other drug use prevention web-based computer tailoring programs in adolescents, with promising results. For example, a randomized controlled trial to prevent alcohol consumption in adolescents, with a theoretical and methodological design similar to that proposed in this article. The intervention successfully reduced binge drinking among 15 and 16-year-olds [40]. In Spain, the cross-cultural adaptation of the intervention designed by Jander et al. has also been carried out with efficacy data for the reduction of alcohol consumption reported [38, 42].

### The study design

The objective of this article is to describe the protocol for the design, the implementation, and the evaluation of a computer-tailored web-based intervention (Alerta Cannabis) aimed to prevent cannabis use among Andalusian adolescents.

## Materials and methods

**Fig. 2:**
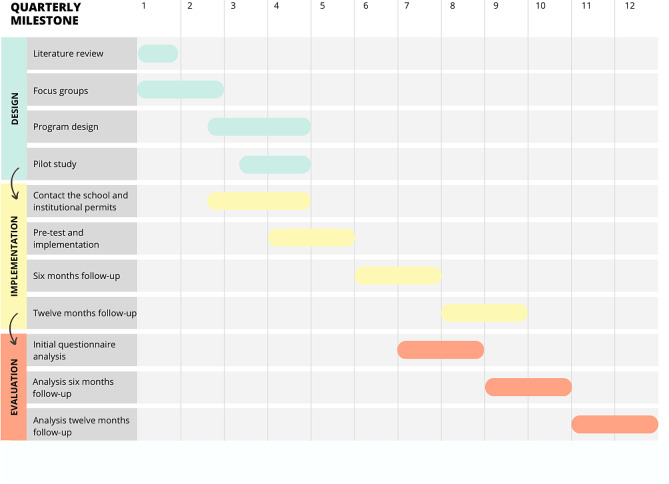
Schedule for the design, implementation, and evaluation of the Alerta cannabis program

This project is based on a cluster-randomized controlled trial in which the new program Alerta Cannabis is tested. This section will be divided into three phasesas described in the following figure (Fig. 2). It has been estimated that to carry out this project 36 months are needed.

### Intervention development

#### Design of the intervention

The intervention will be based on previous studies carried out by the research team [40, 42–44], specifically, it will be based on the Alerta Alcohol program [41]. The development of the content of the intervention will be also based on in-depth interviews with young people in rehabilitation treatment for cannabis use and focus groups with adolescents who are not habitual users of cannabis [45]. A review of the literature will also be carried out to determine the success factors of programs with similar characteristics in cannabis prevention. In addition to being based on scientific evidence, the content of the intervention will be culturally adapted through focus groups with students of similar ages to the target population to learn about the circumstances surrounding cannabis use and the factors associated with cannabis use in Spain (using the I-Change model as a reference). For this qualitative phase, the team follows the COREQ criteria (Consolidated Criteria for Reporting Qualitative research) [46]. Finally, once the content of the intervention is developed, a focus group will be held with experts in prevention to validate it. An advisory and research team will also be consulted in the development of an online intervention for web content development. To finish this stage, a pilot study of the web intervention will be carried out with different class groups to confirm its feasibility and acceptance by the students (feasibility test) [47].

#### Intervention characteristics

This program will be implemented during the academic year during school hours. It is intended for adolescents between the ages of 14 and 18. It consists of four half-hour sessions given once a week or every two weeks by the professors, except for the first, where, in addition to the professors, a member of the research team will be present face-to-face or online, to explain what the course consists of program and how to use the application. Teachers will previously receive all the information about the program through the school counselor, they will even be able to register and use the platform if they want to explore it beforehand. The program is designed to be self-administered, individualized, and personalized, and is very intuitive. The Alerta Cannabis intervention will consist of preventive messages and personalized information on the benefits of not using cannabis, based on the I-Change model. In the first intervention session, the knowledge and risk of cannabis use will be addressed (level of danger, susceptibility, and severity), as well as the pros of cannabis use. In the second session, the cons of cannabis use (emphasizing the negative aspects of its consumption to foster an attitude of rejection of it) and the social model will be worked on (helping adolescents to choose the models they consider most appropriate and encouraging them to seek the support of friends and family who do not use cannabis). In the third session, social norms (helping adolescents to deal with the perceived approval of cannabis use in their environment as well as to choose real relationships that can help them avoid their use) and social pressure (avoiding the influence of people who consume cannabis in their environment) will be worked on. The fourth session is a reinforcement session in which all the aforementioned concepts are worked on and, in addition, the challenge of not consuming cannabis will be proposed to the participants. To evaluate the challenge, the participants who accept it will receive an e-mail asking them to answer whether or not they have been able to overcome the challenge. In all sessions, skills (self-efficacy and action plans) will also be worked on to reject cannabis.

Before starting the first session of the intervention, participants will have to complete an initial questionnaire (pre-test) and two follow-up questionnaires (post-test at six months and twelve months).

### Implementation phase

#### Study design

**Fig. 3:**
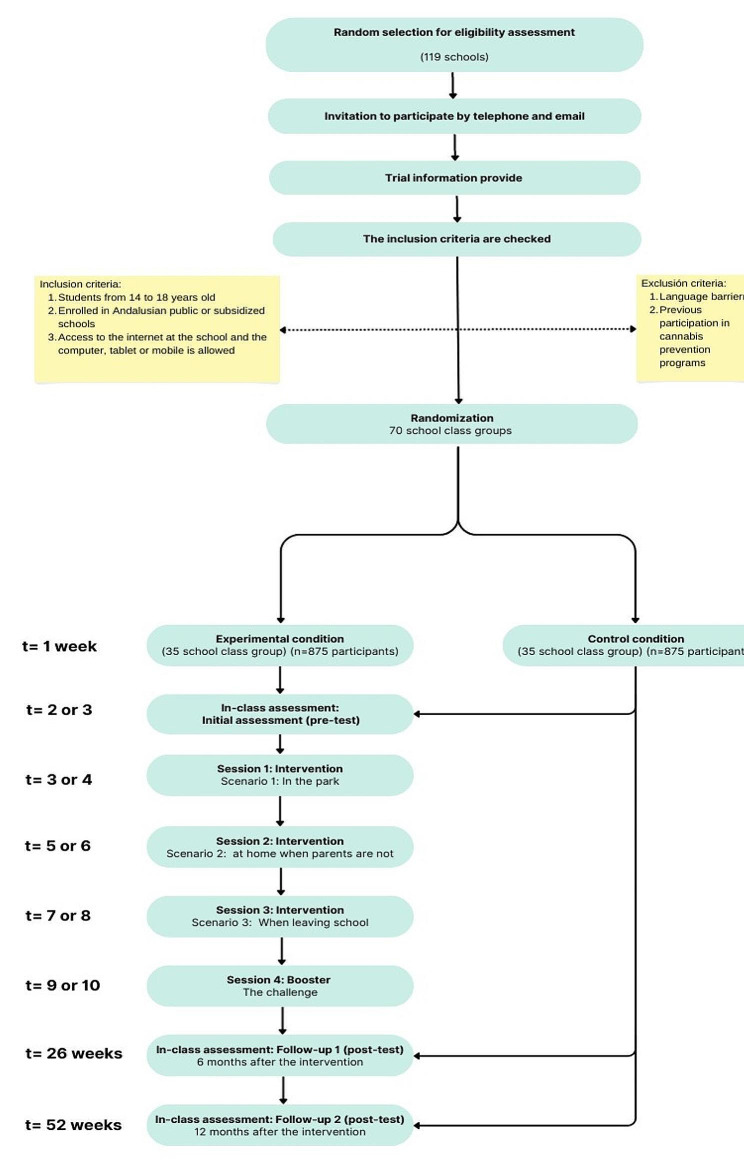
The flow diagram of the intervention describes the steps followed to carry out the intervention, as well as the two study conditions. Sessions for each condition will be also described. The sessions are held at the school and a teacher is present

A two-arm cluster-randomized controlled trial (CRCT) will be designed, with an experimental condition (EC) and a control condition (CC) condition will be randomized at the school level. An initial assessment (pretest) and two assessments (post-test) will be performed at six and twelve months. The allocation ratio will be 1:1 using an online randomization system [48] (Fig. 3). Randomization will be performed by a member of the research team. Randomization will be carried out in batches, since after previous studies we have verified that not all schools confirm their participation in the program within the stipulated period, delaying the start of the rest of the schools in the program. Two school recruitment periods will be stipulated to reach the desired sample size within the same academic year. Participants will be blinded, only knowing that they will be participating in a cannabis prevention project.

#### Participants

The target population will be students between the ages of 14 and 18 enrolled in the third and fourth years of Compulsory Secondary Education (CSE), the first year of high school or intermediate training cycles of publicly affiliated Secondary Education Institutes (SEI) in Andalusia, equivalent to grades 9, 10, 11 and 12, respectively, in the United States. The inclusion criteria in the study for schools are: 1) Secondary School in Andalusia; 2) having access to the Internet; and 3) allowing the use of computers, mobile phones, or tablets to carry out the activity. The exclusion criteria for the students are: 1) having previously participated in a specific program for the prevention of cannabis use 2), having difficulties in the language of the program (Spanish) 3), not wanting to participate or not having parental consent to participate.

#### Sample size

The GRANMO online calculator [49] was used to determine the sample size. It is estimated that after the intervention, the prevalence of cannabis use in the last 30 days will be reduced by 10% [42]. It is estimated that 875 subjects are needed in EC and 875 in CC to find a statistically significant difference between the two proportions of cannabis use. We assumed an intracluster correlation of ρ = 0.2, a minimum of 25 patients for each practice, and a worst-case control rate of 50%. Under these assumptions, we anticipated a power of 87% to detect a difference of 15% in rates between the two groups with α = 0.05 with 60 practices for each intervention group for monitoring accumulating data to protect patients in the trial and future patients.

#### Selection and collection of samples

Contact will be made with all Andalusia schools that have students between the aged of 14 and 18 in their classrooms. According to the data provided by the Education Webpage of the Junta de Andalucía [50] (using the filters secondary education, intermediate vocational training, basic vocational education, and baccalaureate) 119 provincial capital schools can be approached. A member of the investigative team will call all schools. All information provided by telephone will also be mailed to schools interested in participating (program manual, manual summary, application management document, resolution of the Ethics Committee, active consent for parent’s form). This parent-consent form must be signed by parents through iPasen [51], which is the mobile application of the Department of Education and Sports of the Junta de Andalucía that allows communication between different members of the educational community (legal guardians, students, management positions and teachers). Some school nurses also assisted in recruiting schools, during the second batch of recruitment.

Weekly, members of the team will call the counselors of these centers to obtain their definitive participation. The randomization process will be carried out using the Research Randomizer computer software (Version 4.0) [48]. Over the phone, agreements will be made on the dates for the sessions. During the first session, a member of the research team will be present face-to-face or online, so that the teacher who will be present with the students in the next session knows how the platform works (although previously the teacher has been able to use the platform to find out how does it work).

#### Experimental condition (Alerta Cannabis Program)

Participants will complete the Alerta Cannabis intervention (described in Sect.Intervention characteristics).

#### Control condition

Participants in the control condition only will complete one initial questionnaire (pre-test) and two follow-ups (post-test) at six and twelve months.

### Measurements

#### Sociodemographic Variables

They will include gender (female/male), age (in years), parental educational level (none, primary, secondary, university, not knowing), student’s academic year, religion (Catholic, Protestant/Evangelical, Muslim/Islamic, other religion, and no religion) and nationality (Spanish, other). To assess socioeconomic status, the Family Affluence Scale [52] will be used, which consists of six different questions (Does your family own a car, van, or truck? Do you have a bedroom? How many times did your family travel outside of Spain last year on vacation? How many computers (including laptops and tablets, excluding game consoles and smartphones) does your family have? Does your family have a dishwasher at home? How many bathrooms (rooms with a bath/shower or both) are in your house [53–56]. The family Apgar test will be chosen to measure the self-perception of the family’s functional status. It will consist of five questions answered using a three-point Likert-type scale, assessing adaptability or mobilization of resources (Are you satisfied with the help you receive from your family when you have problems? ), participation or cooperation (Do you talk at home about the problems?, What do you have? ), development or growth (Are important family decisions discussed together at home? ), resolve or ability to spend time with a family member (Are you satisfied with the time you spend with your family?) [41].

#### Primary and secondary results: cannabis use and intention to cannabis use

We will measure the frequency and intention of cannabis use. The frequency of cannabis use will be measured with the questions used by the ESTUDES survey [57], which include the days the adolescent has consumed cannabis (hashish/marijuana) in their life, in the last 12 months, and in the last 30 days. In addition, the number of joint consumptions per day will be measured in the last 30 days. We will ask two questions to measure cannabis use intention: Do you intend to use cannabis in the future? Do you intend to use cannabis next year?

#### Motivation factors

To study the motivational factors associated with cannabis use, we based ourselves on previous studies by the team [25].

The advantages and disadvantages of cannabis use will be explored through nine items scored on a five-point Likert scale (1 = strongly agree, 5 = strongly disagree). For example, marijuana use produces family problems (advantage), or cannabis use relaxes me (disadvantage).

Regarding social influences, social modeling will be assessed by asking participants how often people in their environment (i.e., parents, brothers/sisters, friends) use cannabis (1 = never; 5 = always). The social norm will be assessed by asking participants what people around them (that is, parents, brothers/sisters, friends) think about whether the adolescent should use cannabis (1 = definitively should not use cannabis; 5 = definitively should use cannabis). Social pressure will be assessed by asking participants if they have felt pressured to use cannabis by people around them (that is, parents, brothers/sisters, friends) about whether or not they should use cannabis (1 = never; 5 = always). For example, I have felt pressured to use cannabis for my sister.

Self-efficacy will be measured with ten items. Each item assesses whether participants feel confident about not using cannabis in a certain difficult situation (situations that would normally trigger cannabis use, for example, “How difficult or easy is it for you not to use cannabis, if you are at a party and your friend offers you a joint”?). We use a five-point scale (1 = very difficult; 5 = very easy).

### Ethics procedure and approval

The voluntary nature and confidentiality of the data provided by the participants are guaranteed, based on Royal Decree-Law 5/2018, of 27 July on the Protection of Personal Data and the European Data Protection Regulation [58]. Informed consent will be requested both from parents and from the adolescent to participate in the project. This study has been designed in accordance with the Declaration of Helsinki and has the approval of the Andalusian Bioethics Committee (Code 2162- N -20) and the approval of the data management protocol for Alerta Cannabis by the University of Seville.

## Evaluation phase

### Effectiveness and process evaluation

General descriptive statistics will be used to describe the baseline characteristics of the participants. Since the students will be nested within a class in the study and classes will be nested within schools, to examine the predictors of dropout versus non-dropout, a multilevel logistic regression analysis will be conducted. In addition, a multilevel approach will be used to assess the effects of the intervention on the behavior of cannabis use. The first level will be repeated measures within the participants (baseline and two follow-up measurements), the second level will be the student, the third level is the class, and the fourth level is the school.

Baseline cannabis will use behavior and the demographic variables described above will be included as covariates. To select significant predictors and interactions, a backward deletion procedure (a = 0.05) will be used, with the restriction that predictors will not be removed from the model if they were involved as terms.

To study the predictors of adherence, we will also analyze the associations between the potential characteristics (i.e., gender, age, academic course, religion, nationality, family wealth score, Apgar score, and cannabis use at baseline) on the one hand, and participation in the intervention (i.e., adherent or not) through the number of sessions attended by participants at schools on the other.

Finally, for the process evaluation, a descriptive analysis will be performed using chi-square tests to examine differences between males and females and between cannabis users and non-users. We use SPSS Statistics for Windows, version 26.0 (IBM Corp.), for these analyses.

## Discussion

This study is the first to be conducted to design and evaluate an online cannabis use prevention program exclusively in Spain. Cannabis use in adolescents is a public health problem [1] that needs to be addressed through school-based prevention. Several factors are related to its use; a comprehensive socioecological model is needed to understand the various determinants and how best to target preventive activities for adolescents. In the current project, the I-Change Model [24] will be therefore used to develop an interactive and personalized eHealth program for the prevention of cannabis use: Alerta Cannabis, which used principles from earlier similar models targeting smoking prevention [42] and alcohol prevention [41, 44]. To optimize use and implementation, the program will be developed using principles of co-creation, including opinions of experts in prevention, experts in computer engineering, school personnel, as well as adolescents and (former) cannabis users. In Spain, there is a legislative regulation to promote drug prevention in the classroom [59]. Law 4/1997 on Prevention and Assistance in the field of drugs gives special relevance to prevention and frames it within a broad and intersectoral context, including both problem and the causes that determine it. Therefore, schools are obliged to offer these types of activities, which require the collaboration of their teaching staff, implying an extra effort on their part. To help in these training activities, in Andalusia, there are school nurses who perform tasks focused on caring for the health of students and the rest of the school community. To this end, they carry out training activities in the field of health, both for students and for parents, teachers, and other school workers.

A possible limitation of this study could be related to the exclusion criteria related to the prior participation in a specific program aimed at preventing cannabis use, which was assessed among the participant schools during the selection process. However, this criterion does not account for the potential influence of other health promotion or prevention programs within the school environment. As stated in the introduction, In Spain, the school prevention system encompasses various programs, that although not directly linked to cannabis prevention, have demonstrated evidence of impacting cannabis use. Therefore, it is challenging to control for their influence. Furthermore, comparing selected schools based on the number, type, and extent of their prevention programs poses a challenge due to the high variability between regions and within schools. In addition, although there are other possible factors that could influence cannabis use, such as the use of other substances, mental health issues, or other life stressful events, we made the decision to focus solely on general sociodemographic factors to avoid overwhelming adolescents during the completion of the questionnaire. However, we recognize that these factors could act as confounders, and their consideration would be valuable in understanding and explaining why some adolescents may respond more positively to the tested intervention.

In conclusion, it is imperative to continue promoting a culture of evidence-based prevention and to develop efficacy evaluation protocols for prevention programs. By addressing these challenges and conducting further research, we can advance our understanding of effective strategies for substance abuse prevention in school settings. The findings will contribute to the development of future interventions to prevent cannabis use in adolescents. Providing an analysis of the effect and process of a specific program to prevent cannabis use, since the prevention programs of the service portfolio of the Andalusian health system to date have not been evaluated in terms of effect. This program could be a helpful tool for school nurses [60] in charge of implementing prevention programs in the Andalusian context. If the program proves to be effective, the ultimate goal would be national and eventually international implementation.

## Data Availability

This paper is a study protocol so there are not direct data generated. Nonetheless, information on project development can be addressed through correspondence author (mbvillalbba@us.es).

## Declarations

### Registration

ClinicalTrials.gov ID: NCT05849636. Date of registration: March 16, 2023.

## Abbreviations

EC: Experimental condition
CC: Control condition
CRCT: Cluster randomized clinical trial
WBCT: Web-based computer-tailored interventions
CSE: Compulsory Secondary Education
SEI: Secondary Education Institutes
